# Astaxanthin: A Potential Mitochondrial-Targeted Antioxidant Treatment in Diseases and with Aging

**DOI:** 10.1155/2019/3849692

**Published:** 2019-11-11

**Authors:** Mónika Sztretye, Beatrix Dienes, Mónika Gönczi, Tamás Czirják, László Csernoch, László Dux, Péter Szentesi, Anikó Keller-Pintér

**Affiliations:** ^1^Department of Physiology, Faculty of Medicine, University of Debrecen, Debrecen H-4002, Hungary; ^2^Department of Biochemistry, Faculty of Medicine, University of Szeged, Szeged H-6720, Hungary

## Abstract

Oxidative stress is characterized by an imbalance between prooxidant and antioxidant species, leading to macromolecular damage and disruption of redox signaling and cellular control. It is a hallmark of various diseases including metabolic syndrome, chronic fatigue syndrome, neurodegenerative, cardiovascular, inflammatory, and age-related diseases. Several mitochondrial defects have been considered to contribute to the development of oxidative stress and known as the major mediators of the aging process and subsequent age-associated diseases. Thus, mitochondrial-targeted antioxidants should prevent or slow down these processes and prolong longevity. This is the reason why antioxidant treatments are extensively studied and newer and newer compounds with such an effect appear. Astaxanthin, a xanthophyll carotenoid, is the most abundant carotenoid in marine organisms and is one of the most powerful natural compounds with remarkable antioxidant activity. Here, we summarize its antioxidant targets, effects, and benefits in diseases and with aging.

## 1. Introduction

The extended human life span over the past decades is causing the world's population to age. Accordingly, the prevalence of chronic diseases, cognitive decline, and disability increases in the aged population [1]. The first theory to explain the cellular and molecular background of aging was the Free Radical Theory of Aging (FRTA), proposed in the 1950's [2], and has become one of the most studied theories. The basic idea of this theory is that reactive oxygen species and free radicals generated during physiological metabolism and arising from reactions to exogenous factors initiate degradation of biomolecules and the progressive accumulation of these cellular damages ultimately results in aging and age-related diseases. Because of increasing inconsistent evidence, it is now accepted that this theory in its original form or its variants cannot alone explain the aging process [3, 4]. Nevertheless, FRTA cannot be rejected in its entirety, since an impressive amount of data indicates that reactive oxygen species- (ROS-) mediated aging phenotypes and age-related disorders (aside from spontaneous errors in signaling pathways and reactions of metabolites, e.g., reactive aldehydes and sugars) [5–10] exist.

In normal metabolic cells, the production of reactive oxygen and nitrogen species (ROS/RNS) from several sources and their removal by antioxidant systems, including endogenous or exogenous antioxidant molecules, are very precisely balanced. Mitochondria play a major role in supporting the redox balance, so maintaining the structural and functional integrity of these organelles is essential for normal cellular function. At physiological concentrations, ROS/RNS are crucial in signaling processes. These ROS/RNS are produced at very low levels, and the damage caused is almost instantly repaired. The antioxidant enzymes within the cells like catalase, superoxide dismutase (SOD), lactoperoxidases, and glutathione peroxidase inhibit the production of such free radicals and thus present a protecting effect on cells maintaining the balance.

Oxidative stress is defined as an imbalance between prooxidants and antioxidants, resulting in macromolecular damage and disruption of redox signaling and cellular control [11]. Prooxidants are factors that help to generate ROS, which in turn destroy cellular macromolecules, i.e., induce protein oxidation, lipid peroxidation, and DNA damage [12]. mtDNA due to its proximity to the site of ROS production was thought to be intrinsically vulnerable [13]. By contrast, antioxidants reduce oxidative stress, since they act to counter or reduce the effects of ROS [14].

Mitochondrial defects have been proposed to contribute to the development of oxidative stress-related diseases [15, 16]. Impaired mitochondrial function has been involved in various human diseases; particularly, it is considered as the main mediator of the aging process and subsequent age-related diseases [17, 18]. If the reactions of free radicals and other oxygen species contribute to accumulation of molecular damages giving rise to aging and age-related diseases, antioxidants should prevent or slow down these processes and prolong longevity. Based on this assumption, a huge amount of studies were established and aimed at estimating the effect of the level of endogenous antioxidants and/or of the administration of exogenous antioxidants on aging and age-related processes and diseases.

The antioxidant's positive effects are verified by the experimental biological observations which have found that long-term administration of vitamin C decreased the isoprostane levels in rats [19]. Vitamin C was shown to decrease the *α*-tocopheroxyl level in membranes or low-density lipoprotein (LDL) and inhibits *α*-tocopheroxyl radical-mediated propagation [20]. The protective effect of vitamin E against oxidative damage was also demonstrated. It is explained by its direct action on a variety of oxygen radicals. The mechanism of action of antioxidants seems to be widespread: they might directly neutralize free radicals, they quench iron which subsequently decrease ROS production, and they decrease the concentration of peroxide and restore oxidized membranes [21]. Antioxidants are considered as a chemical to be able to induce antioxidant gene expression, to prevent LDL cholesterol from oxidation and provide the brain, the heart, and the liver with antiapoptotic protection [22].

Carotenoids have gained special interest during the last decades, due to their strong antioxidant, repairing, antiproliferative, anti-inflammatory, and potential antiaging effects. They can be used to prevent oxidative stress-related diseases and chronic inflammation. Astaxanthin is one of the most powerful carotenoid on the market. In this review, special focus is oriented towards these compounds.

## 2. The Mode of Action of Retinoids

**Figure 1:**
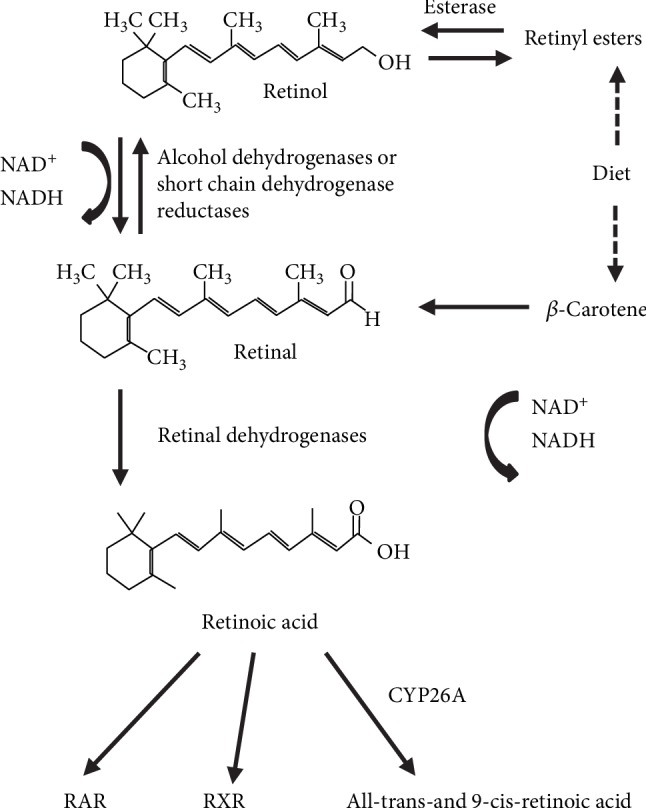
The retinoid conversion cascade.

Vitamin A (retinol) and its derivatives with or without biological activity, collectively referred to as retinoids, are crucial for normal development and homeostasis of vertebrates. The only source of retinoids is through diet (milk, liver, and eggs), because these compounds cannot be synthesized *de novo* [23]. Retinoids have long been appreciated as essential factors for controlling the differentiation program of certain epithelial cells and for their benefic effects on vision, growth, reproduction, and resistance to infection [24]. The predominant natural retinoid in circulation is retinol that is transported at high concentrations (micromolar levels) via the serum bound to retinol-binding protein (RBP), and from there, it can be taken up by any cell for storage or potential conversion into retinoic acid (RA). Once inside, the cell retinol is converted into retinal via a reversible reaction catalyzed by short-chain dehydrogenase/reductases (SDRs). In this reaction, the first step is the conversion to retinaldehyde with the concomitant generation of NADH, and lastly, the formation of retinoic acid by cytosolic aldehyde dehydrogenases (Figure 1). Retinoic acid can bind to and activate nuclear hormone receptors (retinoic acid receptor (RAR) and retinoid X receptor (RXR)). Three different RXR isotypes (RXR *α*, *β*, and *γ*) have been reported so far [25, 26]. RXR*γ* has limited tissue expression including high levels in the brain, anterior pituitary, and skeletal muscle [27].

Retinoic acid, the active metabolite of vitamin A, has notable effects on lipid and energy metabolism by modulating the phosphorylation state of AMPK and Akt [28] and the rate of glucose uptake in L6 myotubes [29]. Treatment of mice with all-trans retinoic acid (atRA) was described to reduce body weight and adiposity independently of changes in food intake, improved insulin sensitivity, and glucose tolerance in lean and obese mice [30, 31] and was found to promote skeletal muscle fatty acid oxidation [32] and irisin production in skeletal muscles [32].

SDRs are ubiquitary expressed and constitute a large protein family involved in the reduction of a variety of substrates. SRP-35 (Sarcoplasmic Reticulum Protein of 35 kDa) was identified using a proteomic approach by Treves et al. [33], and it belongs to the DHRS7C subfamily [34]. In muscle cells, using retinol as its substrate, SRP-35 was proposed as a target to affect glucose metabolism in human patients with metabolic disorders [35]. The biochemical characterization of SRP-35 firmly support that it is a membrane-bound protein with its catalytic site facing the cytoplasm; as a consequence, both its products (retinaldehyde) and NAD(P)H are released into the myoplasm. The generation of NADH in the myoplasm may possess functional signaling relevance; lactate dehydrogenase requires NADH as reducing power to generate lactate from pyruvate, and NADH is available in the mitochondria where it is used to generate ATP. Nevertheless, cytosolic NADH has been shown to regulate the activity of the RyRs, especially in the heart [36, 37].

## 3. Astaxanthin: A Special Carotenoid

Carotenoids, the precursors of vitamin A, are natural pigments supplied with regular highly conjugated *π*-bond systems, providing the natural yellow, orange, or red colors of many vegetables and fruits [38]. Since the structural elucidation of *β*-carotene by the two scientists Kuhn and Karrer in 1930, more than 750 naturally occurring carotenoids have been reported to date [39]. Based on their structure, carotenoids can be compiled into two main groups: (i) the carotenes, also called carotenoid hydrocarbons, which only contain carbon and hydrogen and (ii) the xanthophylls or oxygenated carotenoids that may contain different functional groups (epoxy, methoxy, hydroxy, carbonyl, and carboxyl acid groups) [40, 41].

Besides the most notable carotenoid, *β*-carotene, another carotenoid has been receiving great attention lately: astaxanthin, a marine xanthophyll carotenoid first isolated from a lobster by Kuhn and Soerensen [42]. Astaxanthin is extensively produced by algal species such as *Haematococcus pluvialis* (where it accumulates up to 3.8% on the dry weight basis), *Chlorella zofingiensis*, and *Chlorococcum* and also by the yeast *Phaffia rhodozyma* [43, 44]. Astaxanthin confers the rich pink color observed in various aquatic species including the salmonids and crustaceans and even some nonaquatic species such as the flamingo. Sea creatures cannot produce astaxanthin themselves and must obtain it from their diets, which include zooplankton and krill. Krill oil contains appreciable content of astaxanthin at 0.1 to 1.5 mg/mL depending on processing methods [45]. Krill oil is a superior source of EPA (eicosapentaenoic acid) and DHA (docosahexaenoic acid) which are both long-chain omega-3 fatty acids because the polyunsaturated fats are packaged as phospholipids, which ultimately can be used instantly by our body.

Astaxanthin is a fat-soluble nutrient (it incorporates into cell membranes) with increased absorption when consumed with omega-3-rich seed oil; however, it cannot be converted to vitamin A and therefore cannot support retinol-specific processes such as vision. With its unique molecular structure [46], astaxanthin stretches through the bilayer membrane, providing resilient protection against oxidative stress. It can scavenge and quench ROS and free radicals (superoxide anion, hydrogen peroxide, singlet oxygen, etc.) in both the inner and outer layers of the cellular membrane [46] unlike most antioxidants, which work either in the inner (e.g., vitamin E and *β-*carotene) or the outer side of the membrane (e.g., vitamin C). Astaxanthin derived from the microalgae *H. pluvialis* has been approved as a color additive agent in salmon feeds and as a dietary supplement for human consumption for more than 20 years in dosages up to 12 mg per day and up to 24 mg per day for no more than 30 days in Europe, Japan, and USA [47].

Recent human studies elaborate on the safety perspectives of natural astaxanthin, and so far, no documented negative effects have been found over its 20 years of consumption as a dietary [48]. Clinical studies have found that natural astaxanthin supplementation improved blood flow in humans [49] and enhanced blood rheology by increasing the flexibility of erythrocyte membranes [50].

Astaxanthin emerged in the spotlight because of its potential pharmacological effects, including anticancer [44, 51, 52], antidiabetic [53, 54], anti-inflammatory [55], immune-stimulating effects [44, 52], and antioxidant activities [55–59] as well as neuro-, cardiovascular, ocular-, and skin-protective effects [60–64]. Studies found that astaxanthin reduces the oxidative stress caused by hyperglycemia in pancreatic *β*-cells and improves glucose and serum insulin levels in diabetes [65]. Furthermore, it has been suggested that astaxanthin is a potential therapeutic agent against atherosclerotic cardiovascular disease [66]. In an elegant work, Barros et al. showed that “astaxanthin can directly cross the blood-brain barrier to reach different mammalian brain regions” [64, 67, 68]. Here, we summarize the effects of astaxanthin on metabolism, insulin resistance, and type-2 diabetes mellitus; furthermore, its advantages on muscle performance, recovery, and atrophy, and effects in the central nervous system and the skin will also be discussed.

## 4. The Metabolic Effects of Astaxanthin

### 4.1. Insulin-Mediated Glucose Uptake

**Figure 2:**
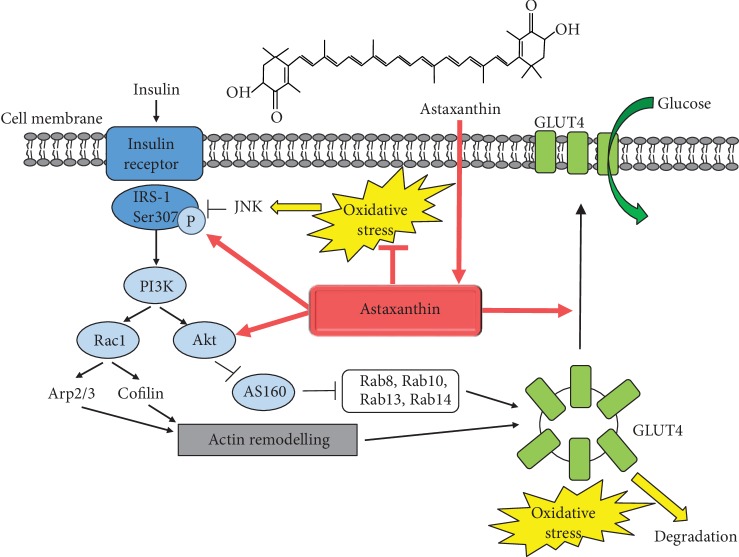
Astaxanthin improves insulin sensitivity and glucose uptake. Schematic representation of the insulin-mediated signaling pathway resulting in the translocation of GLUT4 glucose transporter and glucose uptake. The oxidative stress can lead to insulin resistance by activating various kinases such as JNK, which catalyze the phosphorylation of serine residues in IRS-1 inhibiting its activity and preventing its interaction with the insulin receptor. Furthermore, oxidative stress switches the GLUT4 sorting to the degradation of GLUT4 vesicles. The dietary astaxanthin administration improves insulin sensitivity, IRS-1 activation, Akt phosphorylation, and GLUT4 translocation in skeletal muscle leading to increased insulin sensitivity and a decrease in blood glucose level.

Skeletal muscle accounts for 30-40% of body mass. As the major metabolic tissue of the body, it plays an important role in the whole-body metabolism and homeostasis. In the postprandial state, skeletal muscle tissue is responsible for over 80% of insulin-induced glucose uptake. The molecular mechanisms of insulin-mediated glucose transport are intensively studied. GLUT4 (glucose transporter type 4) is a glucose transporter responsible for glucose uptake into adipocytes and muscle tissue. The GLUT4 vesicles are mainly found perinuclearly at a basal state which are translocated to the plasma membrane by insulin-regulated vesicular traffic leading to glucose transport into the cells and a simultaneous decrease of blood glucose. Importantly, in case of insulin resistance and type-2 diabetes mellitus, the amount of GLUT4 is decreased [69] and its translocation is impaired [70]. The insulin receptor signaling involves the insulin receptor substrate- (IRS-) 1-mediated activation of PI3K (phosphatidylinositol-3-kinase), resulting in Akt2 activation, inhibition of the Akt2 substrate AS160 (Akt substrate 160, a GTPase-activating protein for Rabs), and consequently, the activation of Rab8a and Rab14 GTPases in muscle cells [71]. The PI3K can also activate the Rho-family GTPase Rac1 that is involved in the remodeling of a cortical actin network by regulating the Arp2/3 complex and cofilin influencing GLUT4 translocation [72]. The joint activation of the PI3K-mediated Akt2/AS160 and Rac1 signaling pathways is necessary for the translocation of GLUT4 (Figure 2).

### 4.2. Oxidative Stress and Insulin Resistance

Insulin resistance is a definition for insufficient response of tissues to the effect of insulin, resulting decreased insulin-mediated glucose uptake into the skeletal muscle, increased hepatic glucose production in the liver, and impaired suppression of lipolysis in adipose tissue. The development of insulin resistance is an intensively studied, complex, and not fully known process. Numerous papers have reported that the mitochondrial dysfunction is linked to insulin resistance and type-2 diabetes mellitus [73–75]; however, it remains unclear whether perturbations in mitochondrial functional capacity are causes, consequences, or key contributors to insulin resistance [74, 76]. Mitochondria are the major sources of reactive oxygen species. Insulin resistance is characterized by inefficient mitochondrial coupling, low level of ATP, and the formation of excess amount of ROS despite the normal to high oxygen consumption [73, 75]. Mitochondrial dysfunction was first described in glucose intolerance in 1975 [77]. Several studies suggested that the loss in mitochondrial content and/or function and consequently the decreased mitochondrial oxidative capacity lead to insufficient lipid oxidation, accumulation of lipid excess resulting in the development of insulin resistance [74]. The accumulation of ROS can activate various kinases such as PKCs (protein kinases C), IKK *β* (inhibitor *κ*B kinase-*β*), JNK (c-Jun N-terminal kinase), and p38 MAPK (mitogen-activated protein kinase), which induce the phosphorylation of serine residues in IRS-1 leading to the inhibition of its activity and directing it to proteasomal degradation [74, 78–81]. JNK1 has a crucial role in the development of insulin resistance by inhibiting IRS activity via phosphorylation of Ser307 residue preventing its interaction with the insulin receptor [82, 83]. Furthermore, oxidative stress inhibits the retromer function in a casein kinase-2- (CK2-) dependent manner leading to the sorting of GLUT4 vesicles to lysosomes for degradation [84] (Figure 2).

### 4.3. Astaxanthin Treatment, Insulin Sensitivity, and Muscle Metabolism

Astaxanthin accumulated in skeletal muscle and was shown to reduce hyperglycemia and ameliorate insulin secretion and sensitivity by improvement of glucose metabolism and *β*-cell dysfunction by GLUT4 regulation. Astaxanthin administration increased the translocation of GLUT4 transporter and also insulin-dependent glucose uptake in line with increased phosphorylation of IRS-1 tyrosine and Akt and a decrease in JNK and IRS-1 Ser307 phosphorylation in L6 muscle cells [85]. Inflammatory cytokines (e.g., TNF*α*, tumor necrosis factor *α*) and fatty acids are released from adipose tissue and serve as the major contributors to induce insulin resistance [85, 86]. Palmitate generate ceramide which triggers mitochondrial oxidative stress and insulin resistance [87], and the role of TNF*α* in the generation of insulin resistance was also shown [86]. Importantly, astaxanthin treatment restored TNF*α*- and palmitate-induced insulin resistance and decreased ROS generation of L6 muscle cells [85].

Astaxanthin treatment (8 mg/day, 8 weeks) was effective in patients with type-2 diabetes mellitus: reduced visceral fat mass, serum triglyceride, very-low-density lipoprotein cholesterol concentration, and decreased systolic blood pressure. Furthermore, astaxanthin significantly reduced the fructosamine and plasma glucose concentration [54]. In a metabolic syndrome model SHR/NDmcr-cp (cp/cp) rats where spontaneous hypertension, obesity, hyperinsulinemia, and mild hyperlipidemia evolved, the astaxanthin treatment (22 weeks) ameliorated features of metabolic syndrome: improved insulin resistance; decreased fasting blood glucose, homeostasis of insulin resistance (HOMA-IR), triglyceride, and fatty acid levels; and induced a significant reduction of arterial blood pressure and the size of fat cells in white adipose tissue [88]. Astaxanthin ameliorated high-fat, cholesterol and cholate diet-induced glucose intolerance and reversed hepatic inflammation and fibrosis in C57BL/6J mice [89]. Moreover, astaxanthin administration in a type-2 diabetic db/db (leptin receptor mutated) mice improved the intraperitoneal glucose tolerance test and protected pancreatic *β*-cells against glucose toxicity by reducing blood glucose concentration and hyperglycemia-induced oxidative stress [65]. Insulin resistance can also be detected in another animal model (high fat and high fructose diet-fed mice), where the astaxanthin treatment improved their insulin sensitivity parameters [53]: lowered insulin and glucose levels in the plasma, ameliorated insulin signaling, and enhanced Akt phosphorylation and GLUT4 translocation in skeletal muscle [53].

The number and function of mitochondria influence the fatty acid utilization of the skeletal muscle. The peroxisome proliferator-activated receptor-*γ* coactivator-1a (PGC-1*α*) is a key transcriptional coactivator playing a role in the biogenesis of mitochondria in the muscle. PGC-1*α* was significantly elevated in skeletal muscle samples following astaxanthin intake, and cytochrome C levels were also increased in mice [90]. Moreover, the levels of plasma fatty acids were decreased after exercise in the astaxanthin-fed mice [90], and the fat utilization of skeletal muscle was improved during exercise in a treadmill running model by activation of carnitine palmitoyltransferase I [91]. Interestingly, PGC-1*α* increases the level of GLUT4 and has multiple roles in the pathogenesis of type-2 diabetes mellitus [92], but the effects of astaxanthin on the PGC-1*α*/GLUT4 pathway have not been studied. It has also been demonstrated that peroxisome proliferator-activated receptor (PPAR), which has a major role in the carbohydrate metabolism, is a novel target for astaxanthin. The antioxidant molecule can bind dose-dependently to PPAR*γ* and act as an antagonist or an agonist depending on the cell context [93].

### 4.4. Protective Effect of Astaxanthin on Diabetes Mellitus Complications

Diabetes mellitus increases ROS production and also decreases antioxidant defense capacity. Reactive radicals are produced in several ways, one source is the activated macrophages and neutrophils. Release of large amounts of ROS leads to oxidative stress of all cell components and induces chronic inflammatory responses [81, 94]. It has also been suggested that carotenoids are capable of protecting the different tissues from the long-term consequences of diabetes including nephropathy, infectious diseases, and abnormalities in the neuronal system and eye. Several reports try to examine and discuss the mechanisms behind the biological effects of carotenoids for the prevention of the complications of diabetes mellitus.

Astaxanthin supplementation markedly reduced the level of inflammation-related proteins COX-2 (cyclooxygenase-2), iNOS (inducible nitric oxide synthase), MCP-1 (monocyte chemoattractant protein 1), NF-*β* (nuclear factor beta) in the liver, and the ROS-induced lipid peroxidation in streptozotocin-induced diabetic rats [95]. In human mesangial cells, astaxanthin prevented the high-glucose exposure-induced elevated ROS production in the mitochondria, so it can have a protective effect against diabetic neuropathy [96]. In human neutrophil cells, astaxanthin prevented the high-glucose-induced ROS/RNS production and improved the phagocytic capacity of the cells [97]. The redox balance in the lymphocytes was ameliorated by astaxanthin application via lowering the activities of catalase, restoring ratio between glutathione peroxidase and glutathione reductase activities and lowering the scores of lipid oxidation in an alloxan-induced diabetic rat model [98]. Inflammation-related neuronal apoptosis leads to learning and memory deficits. Astaxanthin decreased the activity of apoptosis-related molecules (TNF, IL-1, and IL-6) and caspases 3 and 9 in the cortex and hippocampus of diabetic rats improving cognitive deficits [99]. High-glucose concentration-induced superoxide, nitric oxide, and peroxynitrite generation was also reduced by astaxanthin treatment in proximal tubular epithelial cell, which inhibited the nuclear translocation of NF-*κ*B p65 subunit [100]. Oxidative stress is the major cause of renal fibrosis during the progression of diabetic nephropathy. In diabetic (db/db) mice, astaxanthin administration improved the development and acceleration of diabetic nephropathy [101]; it improved experimental diabetes-induced renal oxidative stress and prevented renal fibrosis by upregulating connexin43 and activating the antioxidant Nrf2- (NF-E2-related factor 2-) ARE (antioxidant responsive element) pathway in glomerular mesangial cells [102]. In streptozotocin-induced diabetic rats, 12 weeks of astaxanthin treatment ameliorated morphological changes in the kidney via decreasing the protein expression of fibronectin and collagen IV and through the activation of Nrf2-ARE signaling [103]. The therapeutic effect of astaxanthin and other carotenoids regarding long-term complications of diabetes mellitus has been demonstrated in a review of Roohbakhsh et al. [104].

## 5. Effects of Astaxanthin on Muscle Performance, Recovery, and Atrophy

### 5.1. Effects of Antioxidants and Their Targets in Skeletal Muscle Work

During skeletal muscle work, ROS are generated from either mitochondrial or nonmitochondrial sources. These include NADPH, xanthine oxidases, phospholipase A2, and nitric oxide that originates from NO synthase. During moderate exercise, oxidative balance is kept within physiological limits to minimize the effects of oxidative damage [105]. It contains a complicated antioxidant defense system: antioxidant enzymes like glutathione peroxidases, superoxide dismutase (SOD), thioredoxins, peroxiredoxins, and catalase. They are capable of reducing ROS, while endogenous antioxidant substrates such as glutathione can scavenge ROS/RNS [105]. Physical exercise *per se* has antioxidant effects by enhancing these endogenous antioxidant defenses [106], and in pathological conditions, like diabetes or cancer, this endogenous antioxidant effect is probably the most efficient health-promoting mechanism. Despite this, antioxidant treatment is very popular and widely used in medical treatment and also among individuals doing recreational or professional sports to enhance activity. These treatments can modify skeletal muscle signaling like force production, glucose uptake, insulin sensitivity, ion pump functions, and mitochondrial biogenesis [105]. However, the frequently supplemented antioxidants are usually vitamin C and E which generalized nontarget scavengers of all ROS. This leads to the fact that in some cases, these antioxidant supplementations does not decrease, or may even increase, the incidence of human diseases [107] explaining the urgent need of new, more specifically working antioxidant compounds.

Mitochondria have an essential role in skeletal muscle contraction. This is the place of ATP synthesis, modulates redox status, controls pH, and contributes in physiological calcium ho-homeostasis. Since without ATP and calcium muscle fibers do not contract, any alteration on mitochondrial status could lead to myopathy or muscle-related disease like diabetes. It was shown that chronic exercise increased mitochondrial size and density and its cardiolipin content in type-2 diabetes [108]. In Barth syndrome, which is an X-linked recessive disorder manifesting in muscle weakness and cardiomyopathy, dysfunction of tafazzin (a mitochondrial acyltransferase) reduces cardiolipin content and alters mitochondrial function. Treatment with mito-Tempo (a mitochondria-specific antioxidant) in cardiac myocytes lacking tafazzin normalized its level, decreased mitochondrial ROS production, and increased cellular ATP content [109]. Unfortunately, not all the mitochondrial-targeted treatment have beneficial effects. Recent study shows in Barth Syndrome again that a targeted overexpression of catalase in mitochondria did not prevent the development of myopathy in mice [110]. Next to the pathological conditions, aging also decreases mitochondrial functions. In a special animal model of aging, mtDNA mutator mice (they accumulate errors in their mitochondrial DNA and present subsequent changes in the respiratory chain composition) were treated with the antioxidant SkQ1 (10-(6′-plastoquinonyl)decyltri-phenylphosphonium cation) and the phosphorylation capacity of mitochondria in skeletal muscle was improved [111]. This positive effect of SkQ1 was partly because the treatment restored the cardiolipin amount in the mtDNA mutator mice to a wild-type level.

### 5.2. Effects of Astaxanthin during Physical Exercise and Muscle Injury

During heavy exercise, training and competition elevation of reactive oxygen and nitrogen species evolve causing damage in lipid, protein, and nucleic acid molecules. That is why, special nutritional strategy, like supplementation with antioxidant compounds, is now essential for active individuals and athletes. Based upon mouse exercise experiments, supplementation with astaxanthin can effectively improve the side effects of exercise metabolism and the individual's performance and recovery.

Four weeks of astaxanthin treatment in mice prolonged the running to exhaustion. During exercise, astaxanthin administration facilitated lipid metabolism instead of glucose utilization, which improved the endurance and reduced adipose tissue [91]. The same group showed the effect of astaxanthin on ROS-targeted proteins involved in skeletal muscle metabolism during exercise. They found that the oxidative stress-induced modification of lipid peroxidase carnitine palmitoyltransferase I (CPT I) was reduced with the application of antioxidant astaxanthin [112]. Liu and coworkers [90] also suggested that astaxanthin intake increases the PGC-1*α* level in skeletal muscle leading to the acceleration of lipid utilization by the activation of mitochondrial aerobic metabolism during exercise. In oxidative-type soleus muscle, 45 days of astaxanthin supplementation resulted mitochondrial-targeted action, as the treatment increased glutathione content in the mitochondria during exercise, limited oxidative stress, and delayed exhaustion in Wistar rats [113].

Unlike in exercising mouse model, where astaxanthin supplementation enhanced mainly the utilization of fat and depleted muscle glycogen stores during endurance exercise [114], 4 weeks of treatment did not influence significantly the carbohydrate and fat oxidation rate in exercising humans [115]. In this study, Res et al. also reported that there is no significant effect of astaxanthin supplementation on performance during endurance training not even on longer training time periods or in a higher dose (20 mg/day, 4 weeks) in young, trained individuals [115]. Moreover, a high carotene-containing diet also proved to be effective to moderate some of the negative outcomes of sarcopenia on a low physical performance by reducing DNA damage in aged humans [116]. It has been proved that astaxanthin-containing diet modified the expression level of PGC-1*α*, thereby inducing the mitochondrial biogenesis *in vivo* [90]. It was shown also that the prolonged supplementation has not modified the lipid oxidation in order to spare glycogen stores during training, as it already proved in animal studies, which can be due to the increased fitness levels of the inspected subjects [91]. Krill oil treatment also activated the mTORC1 signaling pathway as it was shown in C2C12 myoblasts; however, in young, untrained, healthy individuals, 3 g of krill oil (0.5 g astaxanthin content) administration during 8 weeks did not elevate significantly the muscle force in resistance exercise [117]. In elder subjects (between 65 and 85 years), astaxanthin-containing (12 mg) diet with additional antioxidative properties (10 mg tocotrienol, 6 mg zinc) significantly improved the performance in endurance training and additionally enhanced the force and muscle mass compared to the control group with placebo and training alone [118]. However, authors did not provide information about which signaling pathways were involved and there was also a lack of data about the chronic effects of the astaxanthin treatment.

A fresh study showed that astaxanthin treatment helps to preserve mitochondrial integrity and function in heat-induced skeletal muscle injury examined in cultured C2C12 cells and isolated rat skeletal muscle fibers. The supplementation prevented mitochondrial fragmentation and depolarization, reduced apoptotic cell death, and increased PGC-1*α* and mitochondrial transcription factor A expression following heat stress [119]. Human investigations have also been carried out to show the effect of astaxanthin application on exercise-induced muscle injury. Eccentric loading was applied for 3 weeks in resistance-trained men, and different markers of muscle injury (muscle soreness, creatine kinase level, and muscle performance) were tested. According to the results, the antioxidant supplementation did not favorably affect the aforementioned markers [120]. In another study, cardiac troponin release was examined after endurance-type exercise in cyclists. In this experiment, astaxanthin treatment had no effect on antioxidant capacity (uric acid, malondialdehyde) and inflammation (high-sensitivity C-reactive protein) markers and did not change creatine kinase release induced by exercise [121]. However, a positive effect of antioxidant astaxanthin was suggested in untrained healthy men, where the supplementation significantly increased carbohydrate oxidation and oxygen consumption during exercise and decreased the plasma insulin level. These results indicate that astaxanthin-rich foods can positively affect aerobic metabolism of carbohydrate and fat during rest and exercise [122].

However, several studies using a variety of animal model of myocardial ischemia and reperfusion demonstrated efficiency of astaxanthin supplementation by reducing markers of oxidative stress and inflammation [123]; one has to consider several aspects with regard the use of antioxidant nutritional supplementation for attenuating muscle injury. These supplements seem to attenuate a certain sign of muscle injury during exercise; however, it is not clear what is the optimal dose and treatment period and whether the effectiveness is specific to nonresistance-trained individuals [124]. It is also urgent to find the best markers of skeletal muscle injury and more suitable analytical methods so that more reliable conclusion can be generated regarding the effect of antioxidant agents like astaxanthin in exercise-induced muscle damage. All the questioned aspects of astaxanthin supplementation on exercise performance and recovery have been collected and discussed in a recent review [125].

### 5.3. Muscle Atrophy Is Ameliorated by Astaxanthin Treatment

Skeletal muscle atrophy can occur in case of physiological and several pathological conditions such as immobilization, aging, chronic diseases (e.g., heart failure and renal failure), or cancer. A correlation between oxidative stress and muscle mass has already been observed, the increased production of reactive oxygen species has important roles in disuse muscle atrophy by increasing protease activation [126, 127], and the activation of oxidative stress pathways in atrophying muscles has been suggested to cause apoptosis. Oxidative stress participates in the activation of lysosomal proteases (e.g., cathepsin L), calcium-activated proteases (calpain), and also the ubiquitin-proteasome pathway during disuse muscle atrophy leading to the activation of proteolysis [126–128]. The effects of antioxidants in disuse muscular atrophy have been investigated [129], and the antioxidant astaxanthin comes to the front as an effective molecule to prevent inactivity-induced muscle atrophy. Astaxanthin supplementation prior and/or during hind limb unloading prevented muscle atrophy in different animal models. Dietary astaxanthin intake for 14 days before and during hind limb immobilization attenuated muscle atrophy in rats and interfere with the increased expression of CuZn-SOD (CuZn-superoxide dismutase), cathepsin L, calpain, and ubiquitin caused by immobilization [130]. In another rat model, the dietary astaxanthin supplementation for 2 weeks prior to unloading and during 7-day long immobilization attenuated soleus muscle atrophy and suppressed myonuclear apoptosis measured by the number of TUNEL-positive nuclei [131]. The capillary number is related to the loading and activity of skeletal muscle [132]; the unloading results in capillary regression. Administration of astaxanthin decreased the ROS production, decreased the level of SOD-1, and increased the expression of VEGF (vascular endothelial growth factor) in the soleus of hind limb unloaded rats [133, 134]; furthermore, the 7-day long astaxanthin administration reduced the capillary regression during unloading [133], while the 2-week long treatment maintained the capillary network near control levels [134]. Interestingly, the 2-week astaxanthin diet had a little effect on soleus muscle mass during unloading; other muscles were not examined in this study [134]. Further study showed that the combinatory treatment with dietary astaxanthin supplementation and heat stress prevented disuse muscle atrophy in the soleus muscle, and this protective effect may be partially due to the higher number of satellite cells (stem cells of the skeletal muscle) [135]. Increased ROS production within immobilization-induced skeletal muscle mediates TGF-*β*1-induced fibrosis via promoting the differentiation of fibroblasts and increasing collagen synthesis, where astaxanthin application attenuated skeletal muscle fibrosis [136]. Based on a recent paper by Liu et al., astaxanthin formulation in combination with a functional training program increased the tibialis anterior muscle size determined from magnetic resonance imaging in the elderly [118].

## 6. Effects of Astaxanthin in the Central Nervous System and the Skin

Oxidative stress is thought to be involved in the pathogenesis and progression of age-related cognitive impairments [137] as well. The high lipid content and metabolic rate make the neuronal system particularly vulnerable to oxidative stress. Mitochondrial damage and dysfunction due to oxidative stress are reflected in age-related neurodegenerative diseases such as Alzheimer's disease, Parkinson's disease, Huntington's disease, and amyotrophic lateral sclerosis [138]. In addition to metabolic failure, the membrane of the damaged mitochondria is impaired, their membrane potentials are lost, and they become permeable resulting in the release of cytochrome c. These processes lead to the activation of caspases that induce apoptosis of neuronal cells.

Dietary supplementation with antioxidant vitamins has shown preventive action against oxidative stress and protected or even reversed the age-related changes in antioxidant activity in the central nervous system [139]. In a recent review, Vina and colleagues [140] demonstrated that the systemic oxidative stress and the cognitive function in Alzheimer's disease patients are inversely proportional. The administration of antioxidants, such as vitamin C or E, has been shown to be effective in reducing the symptoms of oxidative stress and cognitive loss [141]. Astaxanthin has recently gained a lot of interest, because among its variety of health-promoting effects mainly through the modulation of parameters related to oxidative stress but also to inflammation, it is able to penetrate the blood-brain barrier accumulating in the brain and has evidenced positive effects on neurodegeneration as well. The neuroprotective effect of astaxanthin was also published using in vitro and in vivo models. The 1-methyl-4-phenylpyridinium (MPP+) toxin was examined which induces neuronal cytotoxicity. The oxidative stress evoked by this neurotoxin opens the mitochondrial permeability transition pore and subsequently triggers the release of cytochrome c. Astaxanthin increases the activity of SOD and catalase, leading to the inhibition of MPP+-induced ROS generation [142]. Astaxanthin seems to restore brain-derived neurotrophic factor (BDNF) levels in both the brains and the hippocampus in rats, thereby slowing brain aging [143]. Astaxanthin's antineurotoxic effect was also demonstrated in an in vivo mouse model of Parkinson's disease [144]. A recent review summarizes further information on the potential neuroprotective role of astaxanthin [145].

Age-related changes of the skin are thought to be driven by two basic mechanisms: biological aging and exposure to ultraviolet rays (photoaging). Photoaging leads to the degradation of components of the extracellular matrix (collagen, elastin), resulting in wrinkles, pigmentation, and deterioration of the skin texture [146]. UV light initiates production of ROS in the skin. Oxidation of C8 guanine base by ROS, 8-hydroxy-2deoxyguanosine (8-OHdG) is produced, which is a marker of DNA damage [147]. In keratinocytes and fibroblasts, ROS activate cytokine receptors and growth factors that will induce the mitogen-activated protein kinase (MAP kinase) and subsequently activate transcription factors of activator protein-1 (AP-1) [148]. AP-1 potentiates the expression of the matrix degrading enzymes, the matrix metalloproteinases (MMPs), which impair collagen in the skin [146]. Continuous carotenoid administration demonstrated protection against UV light [149], and in particular, astaxanthin supplementation had a positive impact on aging skin status, the skin elasticity was restored, the wrinkle formation was attenuated, and epidermal barrier integrity was preserved [150–153]. A comprehensive review of Davinelli et al. summarizes the role of astaxanthin in skin physiology [154].

## 7. Conclusions

To summarize, the antioxidant astaxanthin has attracted increasing attention as an effective molecule to prevent oxidative stress-mediated and age-related diseases. Astaxanthin has been reported to lower plasma glucose and insulin levels and to improve whole body insulin sensitivity and insulin-stimulated glucose uptake. Based on the reviewed studies, it can act as an insulin sensitizer. Astaxanthin also improved disuse muscle atrophy, and it has a neuroprotective role and can prevent the photoaging of the skin. Several papers showed that the excess amount of ROS is involved in the development and progression of chronic diseases, including the pathogenesis of insulin resistance and type-2 diabetes. Pinpointing the further details of the antioxidant astaxanthin effect can yield translational benefits for people with metabolic disease. However, results of studies on the supplementation of model organisms with antioxidants, vitamins, and other antioxidants are divergent; many studies show no effect or even negative life-prolonging effect. Despite these contradictory conclusions, the beneficial effects of antioxidants are undoubted in pathological cases (e.g., antioxidant deficiency); additional studies are needed to demonstrate a positive correlation between antioxidant administration and longevity/or slowing aging for healthy people.
